# Increased Cardiovascular Risk Associated with Chemical Sensitivity to Perfluoro–Octanoic Acid: Role of Impaired Platelet Aggregation

**DOI:** 10.3390/ijms21020399

**Published:** 2020-01-08

**Authors:** Luca De Toni, Claudia Maria Radu, Iva Sabovic, Andrea Di Nisio, Stefano Dall’Acqua, Diego Guidolin, Salvatore Spampinato, Elena Campello, Paolo Simioni, Carlo Foresta

**Affiliations:** 1 Department of Medicine, Unit of Andrology and Reproductive Medicine, University of Padova, 35128 Padova, Italy; luca.detoni@unipd.it (L.D.T.); iva.sabovic@gmail.com (I.S.); andrea.dinisio@unipd.it (A.D.N.); 2 Department of Medicine, Thrombotic and Haemorrhagic Diseases Unit, Padova University Hospital, 35128 Padova, Italy; claudiamaria.radu@unipd.it (C.M.R.); elena.campello83@gmail.com (E.C.);; 3 Department of Women’s and Children’s Health, University of Padova, 35128 Padova, Italy; 4 Department of Pharmacological Sciences, University of Padova, 35128 Padova, Italy; stefano.dallacqua@unipd.it; 5 Department of Neuroscience, Section of Anatomy, University of Padova, 35128 Padova, Italy; diego.guidolin@unipd.it; 6 Fondazione Foresta ONLUS, 35128 Padova, Italy; samspampi@gmail.com

**Keywords:** perfluoro–alkyl substances, cardiovascular diseases, liquid chromatography-mass spectrometry, membrane fluidity, platelets’ aggregation

## Abstract

Perfluoro–alkyl substances (PFAS), particularly perfluoro–octanoic acid (PFOA), are persisting environmental chemicals showing bioaccumulation in human tissues. Recently, exposure to PFAS has been associated with increased prevalence of cardiovascular diseases (CVDs). However, a causal role of PFAS in atherosclerosis pathogenesis is under-investigated. Here, we investigated the effect of PFOA exposure on platelets’ function, a key player in atherosclerosis process. PFOA accumulation in platelets was evaluated by liquid chromatography-mass spectrometry. Changes in platelets’ membrane fluidity and activation after dose-dependent exposure to PFOA were evaluated by merocyanine 540 (MC540) and anti P-Selectin immune staining at flow cytometry, respectively. Intracellular calcium trafficking was analyzed with Fluo4M probe, time-lapse live imaging. Platelets’ aggregation state was also evaluated with Multiplate^®^ aggregometry analyzer in 48 male subjects living in a specific area of the Veneto region with high PFAS environmental pollution, and compared with 30 low-exposure control subjects. Platelets’ membrane was the major target of PFOA, whose dose-dependent accumulation was associated in turn with increased membrane fluidity, as expected by a computational model; increased activation at resting condition; and both calcium uptake and aggregation upon activation. Finally, exposed subjects had higher serum and platelets levels of PFOA, together with increased aggregation parameters at Multiplate^®^, compared with controls. These data help to explain the emerging association between PFAS exposure and CVD.

## 1. Introduction

Perfluoro–alkyl substances (PFAS), such as perfluoro–octanoic acid (PFOA) and perfluoro–octane–sulfonate (PFOS), are a class of organic molecules characterized by the high degree of fluorination of hydrocarbon chains, and are extensively used in industry and consumer products, including oil and water repellents, coatings for cookware, carpets, and textiles. Properties like grease repellency and flame retardation have led to a widespread use of PFAS in industrial and consumer products since the introduction of the compounds, and they can be found in a variety of consumer goods. In particular, PFAS are non-biodegradable and bioaccumulate in the environment as well as in human tissues [1], with detectable levels in populations all over the world [2]. In fact, PFAS can be absorbed by the intestine or inhaled and, once in the circulation, they may act as endocrine disruptors (ED), leading to severe health consequences, such as neonatal mortality, neurotoxicity, immunotoxicity, and reproductive disorders [3,4]. Furthermore, the elimination of PFAS from the serum of a highly exposed human subjects was estimated to be approximately 5 to 8.5 years, whereas in murine models, it is much shorter [5]. On these bases, in 2015, PFOA was classified as a possible carcinogenic agent (2B group) to humans by the International Agency for Research on Cancer (IARC) [6].

Cardiovascular diseases (CVDs) are the major cause of morbidity and mortality worldwide [7]. Accumulating evidence indicated that exposure to environmental air and pollutants from other sources may increase the risk of CVD [8,9,10]. Epidemiological studies have investigated the potential role of PFAS exposure among the risk factors of CVD, such as glucose homeostasis, metabolic syndrome, body weight, and insulin resistance [11,12,13,14]. In addition, recent studies conducted in nationally representative samples of U.S. adolescents and adults have also shown the positive associations between serum levels of PFOA and PFOS with altered lipid profiles, including total cholesterol, low-density lipoprotein (LDL)-cholesterol, high-density lipoprotein (HDL)-cholesterol, and triglycerides [15]. Furthermore, two recent studies on nationally representative cohorts in the United States reported that exposure to high levels of total PFAS was significantly and positively associated with the prevalence of CVD [16,17]. Finally, a higher prevalence rate of self-reported angina and myocardial infarction was reported in individuals exposed to PFOA in contaminated drinking water [18], whereas a positive association between carotid intima-media thickness, circulating platelets, and endothelial microparticles was documented with increased PFOS serum levels [19,20], suggesting that PFAS are somehow associated with the atherosclerosis process. However, limited conclusions regarding the possible causal role of PFAS in the cellular pathogenesis of atherosclerosis and CVD can be drawn.

In this context, platelets play a pivotal role during vascular injury, being involved in both bleeding prevention and atherosclerosis as a chronic inflammatory disease [21]. In particular, in disease conditions associated with a predominant prothrombotic state owing to the hyper-aggregability of platelets, an abnormal formation of thrombus can occur, resulting in an increased risk of myocardial infarction, stroke, and coronary artery disease (reviewed in the works of [22,23]). Of note, environmental air pollution has been associated with increased platelet activation (reviewed in the work of [24]). As cited above, PFAS are highly persistent in human tissues and biological fluids, achieving blood levels greater 200 ng/mL [4]. In spite of this high level of exposure for blood cell components, the impact of PFAS on blood homeostasis, and in particular on platelet function, has never been investigated in humans so far.

In this study, we investigated the effect of PFOA exposure on platelets’ function. In particular, we firstly evaluated the possible PFOA accumulation in human platelets, by exposing peripheral blood from healthy donors with increasing concentrations of PFOA. Then, we evaluated the effect on major markers of platelets’ activation, such as the expression of P-selectin and calcium trafficking. In addition, we investigated the platelets’ aggregometry, through Multiplate^®^ analyzer. This is a novel method that measures the platelets’ aggregation after the addition of different agonists, overcoming limitations of standard tests performed in platelets-rich plasma [25]. The aggregometry profile was evaluated in subjects living in a specific area of the Veneto region with high PFAS environmental pollution and presenting high serum concentration of PFAS.

## 2. Results

### 2.1. Platelets’ Membrane is the Major Cell-Accumulation Site of Blood PFOA

**Figure 1:**
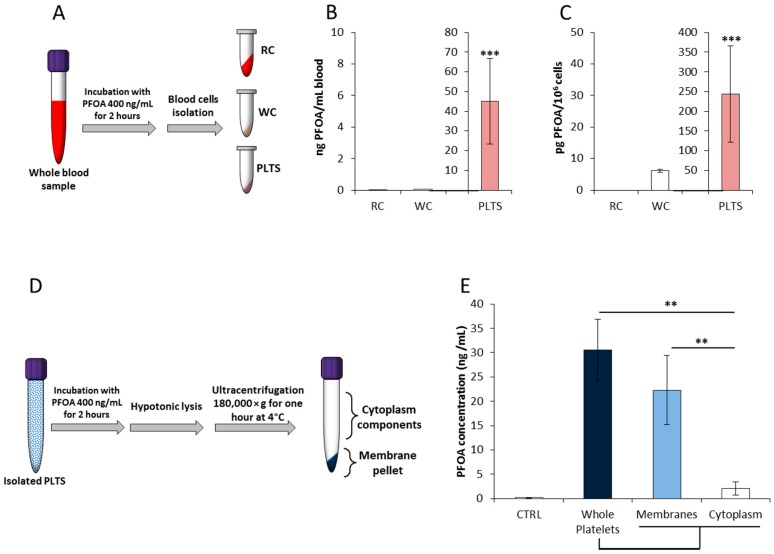
(**A**) Experimental scheme of whole blood incubation with perfluoro–octanoic acid (PFOA) and subsequent isolation of red cells (RC), leukocytes (WC), and platelets (PLTS). The respective cell/platelet content of PFOA was quantified by liquid chromatography-mass spectrometry (LC-MS) and reported as both ng PFOA per mL of whole blood (**B**) and pg of PFOA per million of cell/platelet (**C**). Significance: *** = *p* < 0.001 vs. both RC and WC. (**D**) Experimental scheme of PLTS incubation with PFOA and subsequent isolation membrane and cytoplasm components. The respective content of PFOA was quantified by LC-MS and reported as both ng PFOA per mL of eluate (**E**). In control samples (CTRL), incubation with PFOA was omitted. Significance: ** = *p* < 0.01; *** = *p* < 0.001 vs. indicated samples. All results are reported as mean values of five independent experiments.

Blood levels of PFOA are recognized as the most valuable marker of internal exposure to this chemical, being consistently elevated in exposed populations [26]. In order to evaluate the possible blood-cell target of PFOA accumulation, exposure experiments in whole blood samples were performed as previously described [27]. To this aim, peripheral blood samples from healthy male subjects residing in low exposure areas [4] were incubated with PFOA at the final concentration of 400 ng/mL. Thereafter, erythrocytes, leukocytes, and platelets were isolated by density gradient centrifugation and the cell content of PFOA was quantified by liquid chromatography-mass spectrometry (LC/MS/MS, Figure 1A). Ascertained that basal blood levels of PFOA were below the limit of quantification (LOQ, data not shown), quantification data referred per milliliter of whole blood showed that platelets are the major cell target of PFOA accumulation, accounting for nearly 10% of total blood PFOA content (45.1 ± 21.7 ng/mL; *p* < 0.001 vs. both erythrocytes and leukocytes. Figure 1B). Quantification data referred per million of cells confirmed platelets as the preferential site of accumulation among all blood cell components (< LOQ in erythrocytes; 6.2 ± 0.4 pg/10^6^ cells in leukocytes; 243.9 ± 122.6 pg/10^6^ cells in platelets; *p* < 0.001 vs. both. Figure 1C).

Given the strict similarity with fatty acids, the likely interaction of PFOA with the lipid bilayer of cell membrane has been previously suggested [28,29,30]. Accordingly, we further evaluated the differential distribution of PFOA accumulation between platelets’ membrane and cytoplasm. To this aim, isolated platelets incubated with PFOA at the final concentration of 400 ng/mL underwent hypotonic lysis. Membranes were isolated from cytosol content by ultracentrifugation and subsequent lyophilization (Figure 1D). Quantification of subcellular content of PFOA was then assessed by LC/MS/MS and compared with unprocessed platelets (Figure 1E). Notably, the platelet content of PFOA was essentially confined in the membrane compartment (22.3 ± 7.1 ng/mL membranes vs. 2.1 ± 1.4 cytosol, *p* = 0.003). Taken together, these data suggest that platelets’ membrane is the major cell-accumulation site of blood PFOA.

### 2.2. PFOA Accumulation Alters Platelets’ Membrane Stability at Docking Analysis and the Aggregation Process In Vitro

The possible functional consequences associated with PFOA accumulation in platelets’ membrane were thereafter evaluated.

**Figure 2:**
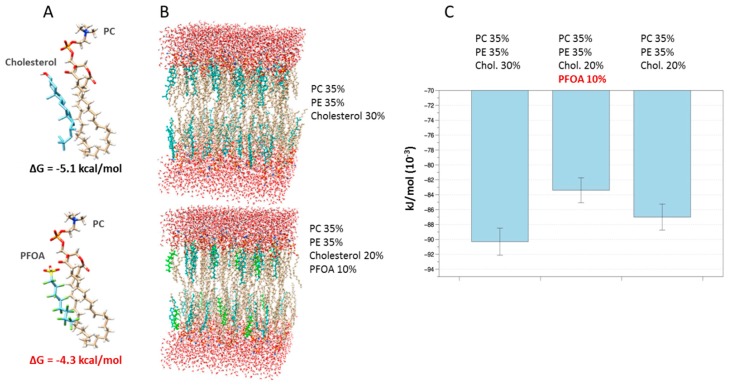
(**A**) Representative models of interaction between phosphatidylcholine (PC) and either cholesterol or perfluoro–octanoic acid (PFOA). Estimated Gibbs free energy (ΔG) changes upon binding are reported. (**B**) Representative models of hydrated bilayers containing 64 lipids per monolayer with 35 water molecules per lipid. In the upper model, a composition of 35% PC, 35% phosphatidyl–ethanolamine (PE), and 30% cholesterol (cyan) was assumed [31]. In the lower model, a composition lipid bilayer involving 35% PC, 35% PE, 20% cholesterol, and 10% PFOA (green) was assumed. In (**C**), the estimated energy of the aforementioned bilayers configuration a reference one, including 35% PC and 35% phosphatidyl–ethanolamine (PE), and another with 20% cholesterol, are reported. Error bars represent standard deviations among different simulations of the bilayers.

A first suggestion on the possible effect of PFOA once it accesses the membrane results from the docking analysis between phosphatidylcholine (PC), the major platelets phospholipid [31], and either cholesterol or PFOA. Data from docking simulations (Figure 2A) suggested that both PFOA and cholesterol can bind to PC, as expressed by their Gibbs free energy (ΔG) changes upon binding, but PFOA exhibiting a lower affinity than cholesterol (predicted ΔG = −4.3 kcal/mol and ΔG = −5.1 kcal/mol, respectively). On the basis of these data, representative simulations of normal platelet membranes, containing 30% cholesterol, and membranes including 10% PFOA and 20% cholesterol, were modelled (Figure 2B). At the equilibrium, bilayers’ configuration including PFOA exhibited an estimated energy about 10% higher than the equilibrium energy of reference bilayers (Figure 2C). Notably, this effect seemed to be partly related to characteristics of PFOA and not only to the reduction of cholesterol. In fact, the model with the same cholesterol reduction, but free of PFOA, exhibited an increase in equilibrium energy lower than that in which PFOA is present. Computational data suggested that accumulation of PFOA in platelets’ membrane is associated with a less stable, more fluid configuration. This hypothesis was probed through different experimental approaches.

**Figure 3:**
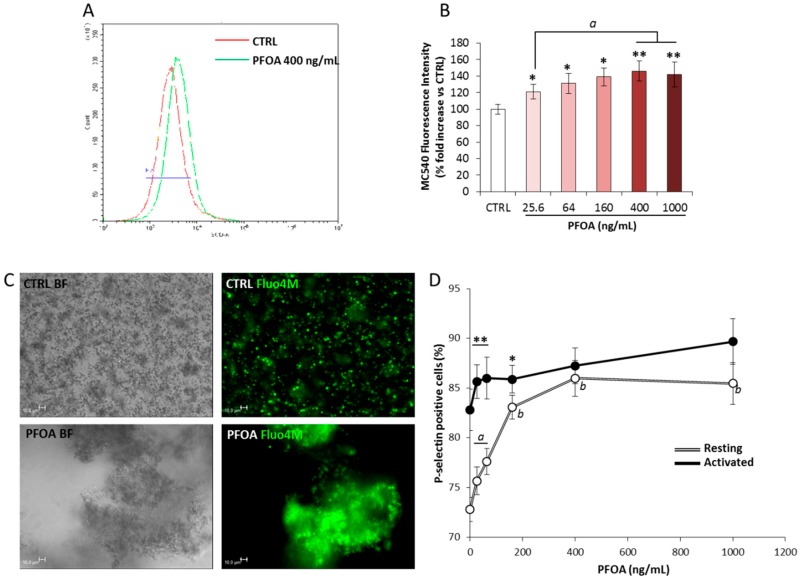
(**A**) Representative histogram plot reporting flow cytometry analysis of resting platelets (PLTS), incubated without (CTRL) or with perfluoro–octanoic acid (PFOA) at the concentration of 400 ng/mL, and subsequently stained with merocyanine 540 probe (MC540). (**B**) Dose response effect on MC540 fuorescence intensity exerted by the incubation of PLTS with PFOA at concentration ranging from 0 (CTRL) to 1000 ng/mL. Significance: * = *p* < 0.05 and ** = *p* < 0.01vs. CTRL, respectively; *a* = *p* < 0.05 among indicated conditions. (**C**) Calcium trafficking images in PLTS not treated (CTRL) and treated with 400 ng/mL PFOA (PFOA). Platelets were activated with 10 μM thrombin receptor activating peptide-6 (TRAP-6). Pictures were acquired after 20 min of time lapse. Platelet morphology was visualized in bright-field images (BF) by differential interference contrast (DIC) (grey-scale images), and calcium uptake was visualized with Fluo-4M probe (green). The images were acquired with a fluorescence microscope (Leica DMI6000CS, 40×/0.60 dry objective) using a DFC365FX camera and LAS-AF 3.1.1 software. Scale bar = 10 μm. (**D**) Platelets’ P-selectin expression after the addition of different concentration of PFOA in resting (white line) or activated platelets (black line) with TRAP-6. Significance: * = *p* < 0.05 and ** = *p* < 0.01 vs. corresponding resting condition, respectively; *a* = *p* < 0.05 and *b* = *p* < 0.01 vs. 0 ng/mL PFOA at resting condition. All results are reported as mean values of five independent experiments.

The possible changes in membrane fluidity owing to the accumulation of PFOA were assessed by the use of the hydrophobic dye Merocyanine 540 (MC540, [32,33,34,35]). Hence, isolated platelets were then incubated with PFOA at concentrations ranging from 0 ng/mL (CTRL) to 1000 ng/mL, stained with MC540, and evaluated by flow cytometry (Figure 3A). Incubation with PFOA was associated with increased values of relative fluorescence intensity (normalized on CTRL, Figure 3B) even at the lowest concentration of 26 ng/mL. Incubation with PFOA at concentration greater than 400 ng/mL was not associated with further increase of the fluorescence intensity, suggesting a saturation process. This evidence was suggestive of an increased platelets’ membrane fluidity associated with exposure to PFOA.

The altered permeability to ions has been claimed as one of the major consequence of increased membrane fluidity in different cell models [36,37]. In particular, increases in intracellular calcium concentration are early upstream events in platelets’ aggregation, resulting in fibrinogen receptor phosphorylation and, in turn, in full platelet activation and aggregate stabilization [38,39,40,41]. Accordingly, the effect of PFOA exposure on calcium trafficking in platelets activated with thrombin receptor activator peptide 6 (TRAP-6) was evaluated by the use of calcium fluorophore Fluo4M, a calcium indicator that increases the fluorescence emission upon calcium binding. The activation with TRAP-6 triggers the release of platelets’ cytosolic calcium, essential for platelets’ degranulation, and thus crucial for platelets’ conformational changes and aggregation [42]. Stimulation with TRAP-6 of platelets incubated with 400 ng/mL PFOA, the lowest saturating concentration observed in experiments with MC540, showed a massive increase of intra-platelet calcium content over time, compared with resting conditions. In addition, platelets’ activation led to the formation of large aggregates (Figure 3C). Differently, platelets not exposing PFOA showed a much lower increase of intracellular calcium concentration, together with the development of very small platelets aggregates.

Finally, the possible activating effect of PFOA on platelets was assessed by evaluating the expression of P-Selectin, in both resting platelets and in platelets activated with TRAP-6, after exposure to PFOA at a concentration ranging from 0 ng/mL (CTRL) to 1000 ng/mL (Figure 3D). Activation with TRAP-6 was associated with a significant increase in the percentage of P-selectin-positive platelets, compared with the CTRL, in all of the conditions tested. However, exposure to PFOA was associated with a significant increase of P-selectin positive platelets in resting conditions, even at the lowest concentration of 26 ng/mL. Resting platelets exposed to PFOA at a concentration equal to or greater than 400 ng/mL expressed levels of P-Selectin statistically not different from platelets stimulated with TRAP-6. Taken together, these data suggest that PFOA alters the platelets’ activation process through the impairment of membrane stability.

### 2.3. Environmental Exposure to PFOA Associates with Hypercoagulability Profile

Finally, the evaluation of whether the altered activation state of platelets exposed to PFOA might result in an impaired aggregation profile in vivo was performed. To this aim, platelets’ aggregation profile was analysed through Multiplate^®^ analyzer in a group of 48 male subjects residing in highly exposed areas of the Veneto region, and compared to that of a control group of 30 male subjects residing in low exposure areas [4].

Demographic and blood parameters of the study cohort are reported in Table 1. In general, blood parameters of the whole study group were within the normal ranges and exposed subjects did not differ from controls for age, body mass index (BMI), platelet and leukocyte count, hemoglobin and mean cell volume levels, and family history of cardiovascular risk factors or cardiovascular diseases. However, exposed subjects showed significantly higher levels of blood PFOA and platelets PFOA (*p* < 0.001 for both). Analysis of the Multiplate^®^ parameters in the study population showed that both exposed and control subjects lay in normal ranges. However, compared with controls, exposed subjects showed higher values for both the arachidonic acid (ASPI) test and TRAP-6 test. No significant differences were observed between controls and exposed subjects in terms of adenosine diphosphate (ADP) test values.

Taken together, these data suggest that exposure to PFOA associates to a platelets’ hyperaggregation profile in young adult male subjects.

## 3. Discussion

In this study, we provide evidence that PFOA distributes unevenly between blood cells, accumulating preferentially in platelets and, particularly, within platelets’ plasma membranes. This primary event associates with altered membrane fluidity and, in turn, with an altered downstream signaling pathway regulating platelets’ activation and aggregation. This hypothesis was confirmed by the evidence of the hyperaggregation profile found in a group of young male subjects residing in highly contaminated PFOA areas in the Veneto region, Italy.

Platelets play an important role in cardiovascular disease both in the pathogenesis of atherosclerosis and in the development of acute thrombotic events. Their importance in coronary disease and in acute coronary syndromes is indirectly confirmed by the benefit of antiplatelet agents in these disorders [43]. Platelet reactivity is altered by a number of environmental factors, such as age, serum cholesterol, diabetes, catecholamine levels, cigarette smoking, obesity, and alcohol consumption. In addition, a role for platelets in the evolutionary phase of the atherosclerotic plaque has been suggested by the observation that platelets can promote foam cell formation even in the absence of hyperlipidemia [44]. However, data from the Framingham Heart Study suggest that these factors play only a minor role, accounting for only 4 to 7 percent of variance. In contrast, estimates suggest that heritable factors play a major role, accounting for 20 to 30 percent of the overall variance in platelet aggregation [45].

The cell membrane plays an important role in cell function and cell–cell communication, and platelets represent an ideal platform for exploring the effects of exogenous factors on membrane fluidity. Upon platelet activation, the asymmetric orientation of phosphatidylserine within the membrane bilayer, essentially represented on the cytosolic surface at resting state, is rapidly disrupted, resulting in a calcium-dependent exposure on the outer surface. This represents a major component of physiological homeostasis because it supports platelet pro-coagulant function [46]. The ratio of membrane cholesterol/phospholipids increases in hypercholesterolemia [47], and has been associated with impaired platelet reactivity to agonists [48,49,50]. In fact, membrane cholesterol content plays important roles in platelet activation and calcium signaling, and in vitro uptake of cholesterol by washed platelets was associated with minor increases of platelet aggregation and secretion after stimulation with standard concentrations of typical agonists [51].

Environmental air pollution has been associated with increased platelet activation (reviewed in the work of [52]). However, substantial evidence has dealt with the possible disrupting role of anthropogenic chemicals released into the environment. This is particularly the case for PFAS, for which significant association with independent cardiovascular risk factors has been frequently reported. In fact, significant association between PFAS exposure and thyroid disorders has been suggested, together with the alteration of sex steroids levels as a possible downstream effect [53,54,55,56,57]. In addition, a recent ecological mortality study reported higher mortality rates for diabetes, cerebrovascular diseases, and myocardial infarction during the period 1980–2013 in municipalities with PFAS contaminated drinking water [58]. Furthermore, the effect of PFOA has also been studied among 1216 subjects from the National Health and Nutritional Examination Survey, where positive correlations with cardiovascular disease and peripheral arterial disease were found [17]. In addition, exposure to PFOA and PFOS is associated with increased total cholesterol and low-density lipoprotein cholesterol (LDL-C) [13,59,60].

So far, it has been shown that PFAS may be incorporated into the lipid membranes, and thus may increase the membrane fluidity [30,61,62,63,64,65], leading to alterations of membrane properties. Most platelet agonists, including thrombin, ADP, and epinephrine, stimulate cell surface receptors that span the platelet membrane, representing a potential target for chemicals that induce alterations in membrane fluidity. This process could explain our observations of altered response to pro-aggregating agonist in platelets from both exposed subjects and controls incubated with PFOA. In the presence of PFOA, platelets increase their cytosolic calcium concentration, which leads to an increase in the membrane expression of P-selectin. P-selectin is a component of alpha and dense granules of platelets and is expressed in the activated platelets. There is evidence that P-selectin is important in the inter-platelets’ aggregation, and subsequently stabilizing the initial platelets integrin α_IIb_β_3_ 3-fibrinogen interactions, thus allowing the formation of large and stable platelets’ aggregates [66]. Wu et al., was the first to indicate the role of circulating soluble P-selectin and its involvement in the thrombotic diseases. Furthermore, human subjects with an increased intima-media thickness have high expression of P-selectin, underlying its involvement in the development of atherosclerosis [67]. Accordingly, the high expression of P-selectin and the elevated presence of calcium after stimulation of normal platelets in the presence of PFOA highly suggest a hypercoagulability state. On the other hand, aggregometry evaluation by Multiplate^®^ identified a hyperaggregation profile in platelets of young adult male subjects exposed to environmental PFOA. Of note, platelet aggregometry showed a higher degree of specificity and accuracy compared with tests performed in platelet-rich plasma, being able to identify a hypercoagulability status in obese patients in spite of unaltered prothrombin time, activated partial thromboplastin time, and fibrinogen levels, as previously described [68]. In particular, exposed subjects showed higher values for the TRAP and ASPI test, compared with the ADP test. This is intriguing because, on the base of the disrupting effect of PFOA on platelet membrane, an undifferentiated effect on all agonists active on platelet effect would be expected. It can be thus hypothesized that the different signaling pathways mediating platelets’ activation display some specific sensitivity to PFOA disruption, representing a possible future target of intervention to prevent the pro-aggregating effect of PFOA.

Although intriguing, these results are clearly preliminary and do not provide a causative link between PFAS exposure and CVDs, thus further longitudinal clinical studies are warranted to clarify these aspects.

## 4. Materials and Methods

### 4.1. Platelets’ Isolation for Flow Cytometry and Immunofluorescence Studies

Platelet-rich plasma was obtained from 10 healthy subjects as previously described [68]. Briefly, blood was centrifuged at 250× *g* for 10 min and PRP was diluted 5:1 with PBS containing 10 mmol/L EDTA (PBS/EDTA) and centrifuged at 14,000× *g* for 1 min to precipitate the platelets. Afterward, the platelet pellet was washed twice with PBS/EDTA and resuspended in Tyrode’s buffer (140 mmol/L NaCl; 268 mmol/L KCl; 0.42 mmol/L; NaH_2_PO_4_ 1 mmol/L; MgCl_2_ 12 mmol/L; NaHCO_3_ 2 mmol/L, CaCl_2_; and 5 mmol/l Dextrose; pH 7.4). Platelet count was determined with CELL-DYN Emerald22 hematology analyzer cytometer (Abbott Diagnostics, Abbott Park, IL, USA). Platelets were resuspended at the concentration of 1 × 10^9^ platelets/mL.

PFOA (Wellington Laboratories, Southgate, ON, Canada) was then added to the platelet suspension at a final concentration ranging from 0 ng/mL (control sample without addition of PFOA) to 1000 ng/mL. Subsequently, samples were allowed to incubate in a thermostatic bath at 37 °C for 2 h under gentle stirring. At the end of the incubation time, the platelets were washed twice with Tyrode’s buffer and immediately used for functional tests, as described below.

### 4.2. Liquid Chromatography-Mass Spectrometry

PFOA levels were measured through reversed-phase (RP) liquid chromatography coupled with triple quadrupole mass spectrometry (LC-MS/MS) Agilent Varian 320 (Agilent Technologies, Santa Clara, CA, USA). Briefly, after dissolving both the lyophilisate of either patient’s sera or the platelet pellet in 400 μL of methanol, the labeled standard of PFOA was added at the concentration of 5 ppb (MPFOA, Wellington Laboratories, Guelph, ON, Canada). The product obtained by mixing these constituents was sonicated for 10 min and subsequently centrifuged at 3600× *g* rpm for 10 min. To test the analytical response and to optimize the calibration curve, standard mixture was used at increasing concentrations of PFOA together with isotope-labeled internal standards (MPFOA) at fixed concentrations. This solution was analysed by LC-MS/MS. Signal was acquired in negative ion mode using an electro spray ion source. Needle voltage was 5000 V, drying gas temperature was 300 °C, drying gas pressure was 22 psi, nebuliser pressure was 55 psi, capillary voltage was set to 40, and CID gas was 1.5 mbar. Mass spectrum operating in negative ion mode and specific transition for each perfluoroalkyl analysed species were obtained using MS software. For 13C PFOA, 417 > 372 was used for quantification, and for PFOA, 413 > 369 transition was used. The Limit of Quantification was 0.2 ng/mL. For the chromatographyc separation an Agilent XDB C-18 column was used (3 × 150 mm, 3.5 micron) and eluents were water 0.1% formic acid (A) and Acetonitrile (B). Gradient was starting with 90%A and in 0.5 min go to 50%A, then in 10 min 100%B and stay isocratic up to 20 min. Flow rate was 0.3 mL/min.

### 4.3. Docking and Membrane Modelling

Three dimensional molecular models of PFOA (cid: 9554), cholesterol (cid: 5997), and phosphatidylcoline (cid: 5497103) were retrieved from the PubChem database (https://pubchem.ncbi.nlm.nih.gov/) as pdb files. The simulation of PFOA and cholesterol binding to phosphatidylcholine was then performed using a docking procedure based on the Autodock Vina algorithm [69] implemented in the UCSF Chimera 1.12 (https://www.cgl.ucsf.edu/chimera/) molecular modeling software.

A computer model of hydrated platelet bilayer was constructed based on available data on platelet plasma membrane composition, indicating phosphatidylcholine (PC) and phosphatidylethanolamine (PE) as the most represented phospholipids (present in about the same amount), and a cholesterol/phospholipid ratio of about 0.5 [31]. Thus, reference models containing 35% of PC and PE and 30% of cholesterol were prepared. Three-dimensional molecular models of PC (cid: 5497103), PE (cid: 5282290), and cholesterol (cid: 5997) were retrieved from the PubChem database (https://pubchem.ncbi.nlm.nih.gov/) as pdb files, and a hydrated bilayer containing 64 lipids per monolayer was prepared using MemGen [70] (http://memgen.uni-goettingen.de), a software capable of setting up systems of heterogeneous lipid membranes. MemGen is not restricted to certain lipid force fields or lipid types, but instead, builds membranes from uploaded structure files, which may contain any kind of amphiphilic molecule. The obtained complex was then further processed by the Yasara software [71] (http://www.yasara.org/minimizationserver.htm) to estimate the equilibrium configuration and its energy. Following the same procedure, variations of the reference model to include 10% of PFOA (PubChem cid: 9554) were also constructed and their equilibrium energy was estimated.

### 4.4. Flow Cytometry Analysis

Surface expression of CD62P (P-selectin, Beckman Coulter, Miami, FL, USA) was investigated by flow cytometry. Briefly, 1 × 10^6^ platelets suspended in 100 μL Tyrode’s buffer were labelled with 5 μL of a monoclonal anti-human CD62P-Phycoerythrin (PE) conjugated antibody (Beckman Coulter, Miami, CA, USA). Platelets were kept in a resting state or activated by the addition of 10 μM TRAP-6 (Roche, France). The samples were incubated for 20 min at room temperature in the dark. As negative control, resting platelets without staining and platelets stained with anti-mouse IgG1-PE conjugated isotype control (Beckman Coulter, Miami, FL, USA) were used. Samples were analysed by CytoFLEX flow cytometry and at least 50,000 events were acquired. The results were analysed using the CytExpert 2.3 software of the instruments (Beckman Coulter, Miami, FL, USA). The mean value of fluorescence intensity subtending the highest 1% of the untreated platelets control curve was considered as the threshold for quantification of platelet activation.

Platelet membranes fluidity was evaluated by merocyanine 540 probe (MC540). Briefly, DMSO-stock solution of MC540 was diluted in platelets’ suspension at the final concentration of 4 μM and incubated for 15 min at 37 °C in the dark. Platelets were finally analyzed by CytoFLEX cytometry, as described above.

### 4.5. Analysis of Platelet Calcium Flux

The release of platelets’ calcium ions provides a sensitive tool to evaluate platelets’ activation and reactivity [72]. Isolated platelets (read above for platelets isolation) were resuspended in 1 mL of Tyrode’s buffer at the concentration of 1 × 10^6^ platelets/mL. Platelets were seeded in a glass bottom dish (Ibidi, Gräfelfing, Germany) and incubated with 10 µM Fluo-4 AM (Thermo Fisher, Monza, Italy), 0.1% (*w*/*v*) Pluronic F-127 (Sigma Aldrich, Milan, Italy). Platelets without and with the addition of 400 ng/mL of PFOA were activated with 10 µM TRAP-6. Platelets were analyzed in time-lapse, with DMI6000CS fluorescence microscope and 40×/0.60 dry objective magnification (Leica Microsystem, Wetzlar, Germany) for 30 min at 37 °C. Images were analyzed in real time using a differential interference contrast (DIC) and fluorescence objectives. Samples were acquired using a DFC365FX camera and processed using the Leica Application Suite (LAS-AF, Wetzlar, Germany) 3.1.1. software (Leica Microsystem, Wetzlar, Germany).

### 4.6. Study Population

This study was performed within the annual screening protocol to evaluate male reproductive health in the high schools of Padua and surroundings (Veneto region, northeast of Italy). The aim of this screening is to early diagnose possible risk factors and diseases of the male reproductive system. Here, we report the findings of 78 subjects aged 18–24 who voluntarily agreed to complete the cross-sectional study between October 2018 and June 2019. Included subjects underwent physical examination, including anthropometric measurements and blood pressure measurements (systolic blood pressure, SBP, and diastolic blood pressure, DBP). A venous blood sample was collected after overnight fasting and stored at −80 °C. Biochemical markers, including cholesterol (total, LDL-C, and HDL-C), were measured by standard biochemical methods in the Central Core laboratory of the University of Padua. During the personal interview, information on socio-demographic characteristics, lifestyle, and medical histories was collected.

The following exclusion criteria were considered: (i) the presence of a prothrombotic condition such as acute infections induced by microorganisms occurred in the previous three months, hormonal therapy, acute or chronic cardiovascular diseases, acquired or inherited thrombophilic condition, severe blood hypertension (≥160/100 mmHg), and diabetes mellitus; (ii) recent surgery and/or cancer; (iii) age ≤18 years; and iv) subjects undergoing anticoagulation and antiaggregation therapy.

Written informed consent was obtained from all subjects, and the study was approved by the Research Ethics Committee of the University Hospital of Padua (N. 2208P). The investigation was performed according to the principles of the Declaration of Helsinki. Participants did not receive any reimbursement. On the basis of the geographical distribution of PFAS pollution, subjects were then grouped on the basis of their residence since birth. On the basis of the degree of pollution, regional authorities (Veneto Region: D.G.R. 2133/2016, Annex A and subsequent modifications (2016)) have defined a highly-exposed area, the red area, which is the one with the highest PFAS levels. Among the 78 subjects included in the study, 48 were resident in the red area (exposure group), and 30 lived outside the exposed area (control group).

### 4.7. Multiplate^®^ Platelet Aggregation Assay

Whole blood was collected from 78 male subjects, with 30 and 48 residing in low and high exposed areas, respectively.

Impedance aggregometry measures were performed by Multiplate^®^ analyzer (Roche Diagnostics International LtdCH-6343, Rotkreuz, Switzerland), adding specific agonists to fresh whole blood samples to start platelets’ aggregation [71]. Test cells of the device incorporate two independent sensors units, each of them consisting of two silver-coated highly conductive copper electrodes. The adhesion and aggregation of platelets to the sensor surface change the electrical resistance (impedance). The increase of impedance is detected by each sensor and transformed to arbitrary aggregation units (AU) plotted against time.

Finally, the results were given as area under the aggregation curve (AUC, AUC*min) and calculated as the mean values of the two curves. Platelet aggregation was stimulated using the following agonists: thrombin receptor (TRAP-6 test), arachidonic acid, checking cyclooxygenase-dependent aggregation (ASPI test), and finally adenosine diphosphate (ADP) test.

### 4.8. Statistical Analysis

The results were expressed as means ± standard deviations (SD) of five independent experiments. Prior to data analysis, the Kolmogorov–Smirnov test was used to check for normality of distribution. Parameters not showing normal distribution were log-transformed. The Levene’s test was used to test the homogeneity of variance among groups. If the homogeneity of variance assumption was violated, the Welch test was performed and the respective *p*-value was reported. Differences between two or more groups were evaluated using Student’s t-test or analysis of variance (ANOVA) for comparison of multiple parameters with Bonferroni correction, respectively. *p*-values <0.05 were considered as significant. Aggregometry data were described as median and interquartile range (IQR) and compared with data of a controls group of healthy male matched for age with cases using the Mann–Whitney U-test. Partial Pearson’s analyses were used to detect significant correlations among PFOA and coagulative parameters. Multiple linear regression was performed to confirm clinical and endocrinological parameters associated with coagulation results. Statistical significance was set at *p* < 0.05 (SPSS version 22.0.0, Chicago, IL, USA).

## 5. Conclusions

In conclusion, in this study, we found that platelet membranes represent a major site of cell accumulation during increased levels of blood PFOA. This evidence associates with an impaired downstream signaling of platelets’ activation and aggregation, likely owing to altered membrane fluidity. Altogether, our results could explain the emerging evidence of an association between chemical sensitivity to PFAS and CVD.

## Figures and Tables

**Table 1:** Demographic and blood parameters in 48 exposed and 30 controls subjects.

Parameters	Clinical Characteristics	Exposed (48)	Controls (30)	*p*-Value
Demographic/blood parameters				
Age (years)	//	18.7 ± 0.6	22.1 ± 1.3	0.102
BMI (kg/m^2^)	//	23.4 ± 3.3	24.2 ± 2.6	0.432
Platelets (10^9^/L)	//	227.8 ± 39.9	230.1 ± 40.4	0.811
Leukocytes (10^9^/L)	//	6.9 ± 1.6	7.2 ± 2.0	0.729
Hemoglobin (g/L)	//	154.1 ± 7.6	148.7 ± 6.3	0.612
MCV (fL)	//	86.0 ± 5.7	83.8 ± 7.1	0.197
PFOA serum levels (ng/mL)	//	128.0 ± 48.5	4.7 ± 2.1	**<0.001**
PFOA platelets level (ng/mL)	//	37.2 ± 15.8	<LOD	**<0.001**
Family history				
Hypertension, diabetes,	Yes	21 (43.8%)	11 (36.7%)	0.638
dyslipidemia	No	27 (56.2%)	19 (63.3%)	
Early familiar CVD	Yes	2 (4.2%)	1 (3.3%)	1.000
	No	46 (958%)	29 (96.7%)	
Cigarette smoking	Yes	10 (208%)	5 (16.7%)	0.772
	No	38 (79.2%)	25 (83.3%)	
Alcohol Consumption	Yes	2 (4.2%)	1 (3.3%)	1.000
	No	46 (95.8%)	29 (96.7%)	
Aggregometry				
ADP test [range] (r.r.= 38–85 AUC)	//	52 [44–62]	48 [38.5–62.5]	0.42
ASPI test [range] (r.r.= 39–79 AUC)	//	67 [57.5–77.0]	55.5 [50.75–58.8]	0.002
TRAP-6 test [range] (r.r 69–117 AUC)	//	100 [91.0–115.5]	87 [82.8–96.5]	0.003

Abbreviations: BMI, body mass index; MCV, mean cell volume; fL, femtoliters; PFOA, perfluoro-octanoic acid; CVD, cardiovascular disease; ADP, adenosine diphosphate; ASPI, arachidonic acid; TRAP-6, thrombin receptor activating peptide-6; AUC, area under the curve; r.r. reference range. Significant *p*-values are highlighted in bold.

## Abbreviations

ASPI	Arachidonic acid
AUC	Area under the curve
CVD	Cardiovascular diseases
ED	endocrine disruptors
LC/MS/MS	liquid chromatography-mass spectrometry
LOQ	limit of quantification
MC540	Merocyanine 540
PC	Phosphatidylcholine
PFAS	Perfluoro–alkyl substances;
PFOA	Perfluoro–octanoic acid
PFOS	Perfluoro–octane–sulfonate
TRAP-6	Thrombin receptor activator peptide 6
ADP	Adenosine diphosphate
